# Long-term functional outcome after a low-energy hip fracture in elderly patients

**DOI:** 10.1186/s10195-019-0529-z

**Published:** 2019-04-11

**Authors:** Stijn G. C. J. de Joode, Pishtiwan H. S. Kalmet, Audrey A. A. Fiddelers, Martijn Poeze, Taco J. Blokhuis

**Affiliations:** 1 Department of Trauma Surgery, Maastricht University Medical Center+, P. Debyelaan 25, 6229 HX Maastricht, The Netherlands; 2 Network Acute Care Limburg, Maastricht, The Netherlands; 3 Nutrim School for Nutrition, Toxicology and Metabolism, Maastricht University, Maastricht, The Netherlands

**Keywords:** Hip fracture, Long-term functional outcome, Mortality incidence, Elderly

## Abstract

**Background:**

The incidence of hip fractures is increasing. Elderly patients with a hip fracture frequently present with comorbidities, which are associated with higher mortality rates. Clinical studies regarding long-term functional outcome and mortality in hip fractures are rare. The aim of this study was to analyse the functional outcome and the mortality rate after a follow-up of 5 years in elderly patients with a hip fracture.

**Materials and Methods:**

This combined retrospective and cross-sectional study included patients aged 65 years or older with a low energy hip fracture who underwent surgery in the Maastricht University Medical Center+, the Netherlands. Data such as demographics and mortality rates were retrospectively collected and functional outcome (i.e. mobility, pain, housing conditions and quality of life) was assessed by a questionnaire.

**Results:**

Two hundred and sixteen patients were included in this study (mean age 82.2, SD ± 7.5). No significant differences were found in pain before hip fracture and after 1-year and 5-year follow-ups. Long-term functional outcome deteriorated after a hip fracture, with a significant increase in the use of walking aids (*p* < 0.001), a significant decrease of patients living in a private home (*p* < 0.001), and a low physical quality of life (SF-12 PCS = 27.1). The mortality incidences after 30-day, 1-year and 5-year follow-ups were 7.9%, 37.0% and 69.4%, respectively.

**Conclusion:**

Long-term functional outcome in elderly patients with hip fractures significantly deteriorated, with an increased dependency for mobility and housing conditions and a decreased physical quality of life. In addition, hip fractures are associated with high mortality rates at the 5-year follow-up.

**Level of evidence:**

Level III, a retrospective cohort study.

## Introduction

In 2012, the incidence of hip fractures was 12.706 in patients aged over 65 years in the Netherlands, and will almost double to 21.218 in 2040 because of the aging population [1]. Worldwide, the incidence of hip fractures varies between 20/100,000 and 574/100,000 in South Africa and Denmark, respectively [2]. The increase in hip fracture incidence is expected to have huge consequences for the current healthcare system, since hip fractures in the elderly are associated with multiple co-morbidities like dementia and delirium, which increase dependency rates and mortality rates [3–8].

Several short-term follow-up studies indicate a poor functional outcome after hip fractures, leading to decreased mobility, less self-dependence in activities of daily living and a decrease in quality of life [9–13]. In line with functional outcome, housing conditions may change as well after hip fracture. In a study by Al-Ani et al., 9% of the hip fracture patients without dementia and 69% with dementia were not able to return to independent living [14]. Of all the Dutch hip fracture patients in 2012, 49% returned to their own living environment, 37% went to a nursing home, 10% went to a sanatorium and 4% died during the hospital admission [1]. However, long-term follow-up data of housing conditions after a hip fracture are unknown.

A decrease in life expectancy is another effect of hip fractures that is often described in the literature. A recent meta-analysis showed a mortality hazard ratio of 5.75 in women and 7.95 in men in the first 3 months following a hip fracture [15]. Furthermore, it indicated an excess annual mortality in 5 years after a hip fracture up to 26%. However, the methodology used consisted of estimations based on extracted data from several studies. Other studies also investigated the excess mortality and found similar results, though in most studies this was also calculated by a prediction model [9–18]. Studies on the long-term mortality rates after a hip fracture remain rare.

Considering the limited evidence of both long-term functional outcome and mortality rates after a hip fracture, the aim of this study was to analyse the functional outcome and mortality incidence rate after a follow-up of 5 years in elderly hip fracture patients. Therefore, we studied the use of walking aids, the intensity of pain, the housing conditions, the quality of life and the mortality rate.

## Materials and methods

To analyse both the long-term functional outcome and the mortality, two different methods were combined. Both a retrospective analysis of a previously collected database [8] and a cross-sectional study were performed. These studies included data of patients aged ≥ 65 years with a low energy proximal femoral fracture [AO/OTA type 31 A (trochanteric fracture) and 31 B (femoral neck fracture)] who underwent surgery in a university medical center in the Netherlands in 2012. Patients with a high energy hip fracture (*defined as motor vehicle and motorcycle accidents, a collision of a moped or bicycle* > *35* *km/h, pedestrian struck by a motor vehicle* > *10* *km/h and fall from 2 times the body height)*, patients with an AO/OTA type 31 C (femoral head) proximal femoral fracture, patients with > 2 fractures and patients not living in the hospital area were excluded. Surgical treatment was performed according to the Dutch Guidelines [19].

Data were collected from a previous retrospectively collected database [8] by one independent researcher. Demographics included age at time of fracture, gender, ASA (American Society of Anesthesiologists) physical status score [20], Charlson comorbidity index [21], time between injury and surgery and type of fracture (trochanteric or femoral neck fracture). During the cross-sectional part of this study, all surviving individuals received a questionnaire via regular mail 5 years after the hip fracture. Data from the questionnaire were gathered by another independent researcher. The questionnaire contained four different items: (1) mobility, the use of walking aids and the patient-reported percentage of mobility level 5 years after trauma compared to the level before trauma; (2) pain, the intensity of pain measured with the numeric rating scale (NRS, 0 = no pain and 10 = worst pain imaginable) [22]; (3) the modification of housing conditions, and (4) the quality of life measured with the Short Form 12 (SF-12) and the patient-reported percentage of average health status level 5 years after trauma compared to the level before trauma [23]. The SF-12 consists of 12 items that assess 8 dimensions of health: physical functioning, role-physical, bodily pain, general health, vitality, social functioning, role-emotional and mental health. The SF-12 measures various aspects of physical and mental health from which physical composite score (PCS) and mental composite score (MCS) can be calculated. A score of 100 means maximum quality of life, 0 means lowest quality of life, and the norm for elderly people was recently reported to be 36.7 and 50.1 for the PCS and MCS, respectively [23]. The use of walking aids, the intensity of pain and the modification of housing conditions were all asked for different time periods: before hip fracture, directly after hip fracture (before surgery) and at 30 days, 1 year and 5 years after surgery. The results of these functional outcome variables were requested at the Dutch central national registry, called the Central Bureau for Statistics (CBS) to compare 5-year follow-up results between the study population and the average 77-year-old Dutch population in 2011. Five-year follow-up data of the average 77-year-old population in 2012 were not available yet.

Patient outcome measures included the 30-day, 1-year and 5-year mortality rates. These data were collected from the medical records and by telephone contact with general practitioners and relatives. The 5-year mortality incidence rate of the average 82-year-old population in 2012 was calculated by the CBS [24].

The medical ethics committee of the university medical center approved this study and written informed consent was obtained from all individual participants included in the study.

### Statistical analysis

IBM SPSS statistics, version 23.0, was used to perform statistical analysis. Descriptive statistics (e.g. frequencies, mean and standard deviation), were used to describe the demographics and the baseline and follow-up characteristics of the elderly hip fracture patients.

Independent sample *t*-tests were used for parametrically distributed continuous data, (e.g. NRS) and *χ*^2^ tests for categorical variables (e.g. use of walking aids and housing conditions). Results are presented as mean ± standard deviation (SD) or as frequencies and percentages. The median with the interquartile range (IQR) were used to describe non-parametric data (e.g. physical and mental SF-12 summary scores). An alpha of 0.05 was set as a level of statistical significance.

## Results

### Baseline characteristics

In the year 2012, 220 hip fractures were treated surgically in the university medical center. Of these, 216 met the inclusion criteria. The population at baseline, of whom 71% were female (*n* = 153), had a mean age of 82.2 (SD ± 7.5) years, had a mean Charlson comorbidity index of 7.0 (SD ± 2.6) and 40.3% (*n* = 87) had an ASA I or II score. Baseline characteristics are presented in Table 1. Fracture type distribution was almost evenly distributed between femoral neck and intertrochanteric fractures. Over 55% were operated on within 24 h of the trauma.

**Table 1:** Baseline characteristics

	Total (*n* = 216)
Female (%)	153 (71.0)
Mean age (SD), years	82.2 (7.5)
*ASA*	
I, II (%)	87 (40.3)
III, IV (%)	129 (59.7)
Mean Charlson score (SD)	7.0 (2.6)
*Fracture type*	
Femoral neck (%)	101 (46.8)
Pertrochanteric (%)	115 (53.2)
Operated within 24 h	119 (55.1)
Operated within 48 h	205 (94.9)

*SD* standard deviation, *ASA* American Society of Anesthesiologists

After 5 years, 66 patients (30%) were alive. The mean age of the survivors was 82 years, and the majority (66%) was female. Only 38 individuals were in cognitive good health, able to respond, and willing to fill out the questionnaire.

### Functional outcome measures

1. Mobility: 5 years after their hip fracture, significantly more patients used walking aids than pre-fracture (56.8% vs 29.7%; *p* < 0.001). Data for walking aid use are presented in Table 2. One patient did not fill out this part of the questionnaire, so 37 respondents remained for the reported functional outcome. According to recent data from the CBS, 34.1% of the Dutch population above 75 years of age is in need of a walking aid [24]. The patient-reported mobility level as compared to the pre-fracture level (100%) showed a decrease to 63.8% (SD ± 36.1) at the 5-year follow-up.

**Table 2:** Use of walking aids during a 5-year follow-up

Walking aid	Before trauma	30 days follow-up	1 year follow-up	5 years follow-up
Without walking aid (%)	21 (56.8)	7 (18.9)	14 (37.8)	11 (29.7)
Walking stick (%)	11 (29.7)	13 (35.1)	11 (29.7)	9 (24.3)
Walker (%)	4 (10.8)	15 (40.5)	11 (29.7)	12 (32.4)
Walking frame (%)	0 (0.0)	1 (2.7)	0 (0.0)	0 (0.0)
Wheelchair (%)	1 (2.7)	1 (2.7)	1 (2.7)	5 (13.5)
Total (%)	37 (100)	37 (100)	37 (100)	37 (100)
2. Pain: Patients did not experience more pain at 1 or 5 years after hip fracture than they did before hip fracture: mean NRS 1.97 (SD ± 2.65), 1.89 (SD ± 2.74) and 1.89 (SD ± 2.72), respectively (Fig. 1). These scores are all under the NRS cut-off point of 3, which means there is no significant effect between pain and general activity, mood, walking ability and sleep [25, 26]. According to data from the CBS, 21.4% of the Dutch population above 75 years of age was obstructed by pain in daily living activities in 2016 [24].

**Fig. 1:**
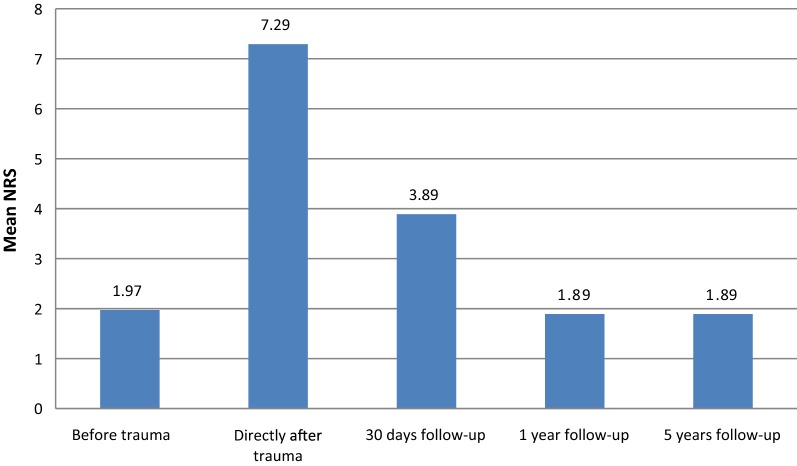
Mean NRS during 5 years of follow-up
3. Housing conditions: Before their fracture, 84.2% of the respondents lived at home versus 60.5% at 5 years after the fracture (*p* < 0.001) (Table 3). Compared to the general population in the same age group, the percentage of people living in a nursing home in our cohort was much higher as well; the overall percentage for 82-year-old inhabitants of the Netherlands living in a nursing home was 6.0% in 2016 [24].

**Table 3:** Housing conditions during a 5-year follow-up

Housing condition	Before trauma	30 days follow-up	1 year follow-up	5 years follow-up
Private home (%)	32 (84.2)	21 (55.3)	30 (78.9)	23 (60.5)
Protected housing (%)	1 (2.6)	2 (5.3)	2 (5.3)	5 (13.2)
Residential care home (%)	4 (10.5)	5 (13.2)	5 (13.2)	6 (15.8)
Nursing home (%)	1 (2.6)	2 (5.3)	1 (2.6)	4 (10.5)
Rehabilitation hospital (%)	0 (0)	6 (15.8)	0 (0)	0 (0)
Hospital (%)	0 (0)	2 (5.3)	0 (0)	0 (0)
Total (%)	38 (100)	38 (100)	38 (100)	38 (100)
4. Quality of life: At the 5-year follow-up, the median Short Form 12 (SF-12) total score was 44.4 (IQR 20.2–66.3). The median PCS and MCS of the SF-12 were 27.1 (IQR 12.5–55.2) and 65.0 (IQR 22.5–80.8), respectively. The mean patient-reported overall health status decreased by 44.7% (SD ± 27.9) in 5 years after hip fracture.

### Mortality

The mortality incidence at 30 days, 1 year and 5 years after hip fracture was 7.9%, 37.0% and 69.4%, respectively (Table 4). The increase between 30 days and 1 year and between 1 and 5 years was significant (*p* < 0.001). A significantly higher mortality rate was found in male compared to female patients at each follow-up (*p* < 0.05). According to the CBS data, the 5-year mortality incidence in the overall Dutch 82-year-old population in 2012 was 22.9% [24].

**Table 4:** Mortality incidence and significance between men and women during a 5-year follow-up

	30 days follow-up	1 year follow-up	5 years follow-up
Mortality incidence (%)	17 (7.9)	80 (37.0)	150 (69.4)
Mortality incidence men (%)	8 (12.7)	26 (41,3)	46 (73.0)
Mortality incidence women (%)	9 (5.9)	54 (35.3)	104 (68.0)
Significance between men and women	*p* = 0.002	*p* < 0.001	*p* < 0.001

## Discussion

The impact of hip fractures in elderly people on mobility and mortality are well known for the 1st year after the fracture has occurred. Only a few studies have analysed the long-term effects. This retrospective study found that hip fractures in the elderly reduced functional outcome in the long term, with an increase in the use of walking aids, a decrease in patients living in a private home, and a low physical quality of life even 5 years after the fracture. Furthermore, hip fractures are associated with high mortality rates at the 5-year follow-up compared to the age-matched general population.

Several studies have reported that significantly fewer elderly patients were walking independently at 1 to 4.9 years after hip fracture [10–12, 27, 28]. Kammerlander et al. found that only 8% of the survivors were able to walk without the use of walking aids at a 4.9-year follow-up [11]. However, this study did not investigate pre-fracture mobility. Pretto et al. showed a decrease in pre-fracture community dwelling patients walking independently from 80 to 57% at a 1-year follow-up [28]. These findings are in line with the results of our study and with the data of the average Dutch population above 75 years of age.

No significant differences in patient-reported pain before and at 1- and 5-year follow-ups were found in this study, but to our surprise the mean pain score for our population, reported before the fracture, at 1 year and at 5 years, was 2.0. This suggests that (some) elderly patients constantly have a low amount of pain during the day and these data are in line with the general Dutch elderly population, where 21.4% are obstructed by pain in their daily living activities [24]. Conversely, following Dihle and Gerbershagen, a NRS of < 3 has no significant effect of pain on general activity, mood, walking ability and sleep [25, 26].

The number of patients living in a private home decreased significantly at the 5-year follow-up (*p* < 0.001). To our knowledge, this is the first long-term follow-up study investigating housing conditions after a hip fracture in elderly patients. Multiple studies have shown a relation between pre-operative housing conditions and short-term functional outcome. As expected, patients living in a private home had a lower mortality rate, had fewer comorbidities and were more independent in daily living activities than patients in a nursing home [11, 28, 29]. The increased number of people moving to a nursing home facility seen in our study may appear to be logical, as the cohort is ageing over 5 years. However, compared to the general population, the percentage living in a nursing home is dramatically increased, underlining the severe impact that hip fractures have on mobility after a long period of time [24].

The quality of life in the current study was investigated using the SF-12. Interestingly, the SF-12 showed two different outcomes. When compared to the average score for older people [23, 30], the physical score was well below average at the 5-year follow-up, whereas the mental score was higher. A study of Moerman et al. has recently found similar results, but only at a maximum follow-up of 12 months [31]. The finding that the physical score is decreased underlines the impact of a hip fracture on mobility, even 5 years after the fracture. As the functional outcome decreases, it is likely that the quality of life declines as well. This is illustrated in our study by the patient-reported average health status, where participants indicated a decrease of 65% in health status over the 5 years after the hip fracture.

Several studies investigated long-term mortality after hip fracture, indicating an excess mortality in the elderly [9–18]. However, most of these studies calculated the mortality by a prediction model, whereas the mortality incidence in our study was compared to the general age-matched population. These data show that the 5-year mortality incidence after a hip fracture is almost 70%, compared to 22.9% for the general population [24]. There is, obviously, a close relationship between the known risk factors for hip fractures and mortality, and the population with hip fractures has more co-morbidities than the general population. Therefore, with the increasing age of the studied population, mortality was expected to increase. Still, the observed difference, 70% in the hip fracture population versus 23% in the age-matched general population, is remarkable. This study also showed a significant dissimilarity between men and women in the mortality incidence, but not as clear as Trombetti et al. found [29]. The cause of this dissimilarity remains unclear.

This combined retrospective and cross-sectional study is one of the first long-term follow-up studies investigating functional outcome in mobility, pain, housing conditions and quality of life. However, some limitations may influence the interpretation of the results. First, the number of survivors is relatively low, and in this group the number of respondents is even lower. For example, patients that were unable to respond because of their cognitive condition were excluded. The risk of selection bias may be considerable, although this study has likely selected the population with the highest functionality. The small sample size, in combination with the selection bias, certainly limits the external validity of the cross-sectional part of this study. Second, the outcome of several variables was compared with data provided by the Dutch central registry. Although this registry is up to date and comprehensive, their data may differ from other countries. Third, the data were obtained from the year 2012. Over the last few years new treatment strategies were imposed, i.e. a multidisciplinary pathway. This may have influenced our outcome, although it is unclear whether such new treatment protocols have any impact on the long-term results. Finally, this study did not investigate the specific causes of decline in functional outcome, since the surviving population was too small.

This long-term follow-up study found that hip fractures in the elderly reduced long-term functional outcomes with an increase in the use of walking aids, a reduction in numbers of patients living in private homes, and a lower physical quality of life. Furthermore, hip fractures are associated with high mortality rates at the 5-year follow-up. These data underline the long-term impact that hip fractures have in the older population, as well as the increase in burden on society that is to be expected in the ageing population in the western world.
